# A hypergraph-based method for large-scale dynamic correlation study at the transcriptomic scale

**DOI:** 10.1186/s12864-019-5787-x

**Published:** 2019-05-22

**Authors:** Yunchuan Kong, Tianwei Yu

**Affiliations:** Department of Biostatistics and Bioinformatics, Emory University, 1518 Clifton Road, Atlanta, 30322 USA

**Keywords:** Gene expression, Network analysis, Hypergraphs, Dynamic correlations, Liquid associations

## Abstract

**Background:**

The biological regulatory system is highly dynamic. Correlations between functionally related genes change over different biological conditions, which are often unobserved in the data. At the gene level, the dynamic correlations result in three-way gene interactions involving a pair of genes that change correlation, and a third gene that reflects the underlying cellular conditions. This type of ternary relation can be quantified by the Liquid Association statistic. Studying these three-way interactions at the gene triplet level have revealed important regulatory mechanisms in the biological system. Currently, due to the extremely large amount of possible combinations of triplets within a high-throughput gene expression dataset, no method is available to examine the ternary relationship at the biological system level and formally address the false discovery issue.

**Results:**

Here we propose a new method, Hypergraph for Dynamic Correlation (HDC), to construct module-level three-way interaction networks. The method is able to present integrative uniform hypergraphs to reflect the global dynamic correlation pattern in the biological system, providing guidance to down-stream gene triplet-level analyses. To validate the method’s ability, we conducted two real data experiments using a melanoma RNA-seq dataset from The Cancer Genome Atlas (TCGA) and a yeast cell cycle dataset. The resulting hypergraphs are clearly biologically plausible, and suggest novel relations relevant to the biological conditions in the data.

**Conclusions:**

We believe the new approach provides a valuable alternative method to analyze omics data that can extract higher order structures. The software is at https://github.com/yunchuankong/HypergraphDynamicCorrelation.

**Electronic supplementary material:**

The online version of this article (10.1186/s12864-019-5787-x) contains supplementary material, which is available to authorized users.

## Background

In the quantitative analysis of high-throughput omics experiments, the gene transcript-, protein- or metabolite-levels of abundance are profiled simultaneously. Examples include high-throughput sequencing of mRNA (RNA-seq) and high throughput mass spectrometry for quantitative analysis of specific cellular proteome or metabolites. The abundance levels of gene, transcripts or metabolites are the outcome of complex biological regulatory networks, in which the links between the levels may be turned on and off in response to certain biological conditions [1–4]. As a result, many correlations are dynamic, shifting between positive, negative and no-correlation states, triggered by certain biological conditions. Such conditions may not exhibit prominent phenotypic changes, e.g. disease/non-disease in case-control studies, but they may be more subtle and often unobservable [5, 6].

Given the profiling data are essentially snapshots of the system, it is challenging to extract higher order relations from the data, such as conditional correlations and changes in variability. To explore patterns in high-throughput expression data, methods that include clustering, dimension reduction and sparse factorization have been proposed. These methods are mostly based on static pairwise relations between the biological units, and do not capture dynamic relations [7, 8].

According to [5, 9, 10], the expression levels of certain genes can be treated as indicators of cellular states, and correlation changes conditioned on such genes are computed to measure dynamic correlations. The involvement of such genes as dynamic correlation condition results in three-way gene interactions, and quantitative measures for the three-way interaction have been developed to quantify the ternary relationship, such as the Liquid Association (LA) statistic proposed by [5], the Modified Liquid Association (MLA) developed by [11], and the *z*-statistic in [12]. These ideas have been demonstrated successfully in practice showing interpretable biological findings at the gene level. Biologically, it is plausible that a single gene may not be a good proxy measure of the underlying condition for the dynamic correlation. However measures involving more than one gene as the conditioning variable is difficult to design, and costly in computation. To address this issue, a method treating the LA relation as latent factor model has been developed, where in stead of using genes as proxy measures, the conditioning variable is estimated from the data [13]. However such an approach can only find dominating signals that control the dynamcic correlations of large numbers of gene pairs. Some critical dynamic correlation may happen among a small group of genes, yet play important biological roles. Hence an unbiased examination of all gene triplets is valuable.

Currently, the existing methods suffer from computational scalability when examining the entire biological system since it is difficult to examine gene-level three-way interactions triplet-by-triplet as the amount of possible combinations is extremely large. Efforts have been made to focus on a smaller number of subsets, by considering consistent LA relations across multiple datasets [14], focusing on subnetwork-level LA relations [15].

Meanwhile, it is desirable to view the complex interactions of individual triplets jointly as a whole, since otherwise it is hard to grasp the dynamic correlation behaviors at the system level. Therefore, an aggregated representation is in need for ternary gene relationships, analogous to the gene co-expression network for the pairwise static correlation relationship. The gap resulted from this problem motivated the work in this paper, where we developed a hypergraph-based approach constructing module-level three-way interaction networks for ternary gene relationship study.

The main difficulty to analyze three-way interaction for an entire system is the extremely large amount of possible triplets at the gene level. For example, for a gene-expression dataset with 20,000 genes, the number of possible combinations would be around 1.33×10^12^. Thus, one can do little when trying to describe the entire system while focusing on gene-level interactions. To resolve the dilemma, we consider a bottom-up approach to bring the ternary relationship to the module level, while preserving partial information of gene-level three-way interactions. This idea allows us to shrink the scale of the system and thus facilitate the aggregated representation. For this purpose, it is covenient to use a hypergraph to present the ternary relations.

A hypergraph *G*=(*V,E*) with *V* the set of vertices (or nodes) and *E* the set of edges (or links), is a generalization of a graph in the sense that an edge can connect any number of vertices rather than just two [16]. A special case when all the edges in *E* connect to a certain number of vertices *k*, the hypergraph is called *k*-uniform hypergraph. Therefore, a traditional graph or network is just a two-uniform hypergraph, and it is obvious that in our case the triplets compose of a three-uniform hypergraph.

**Fig. 1:**
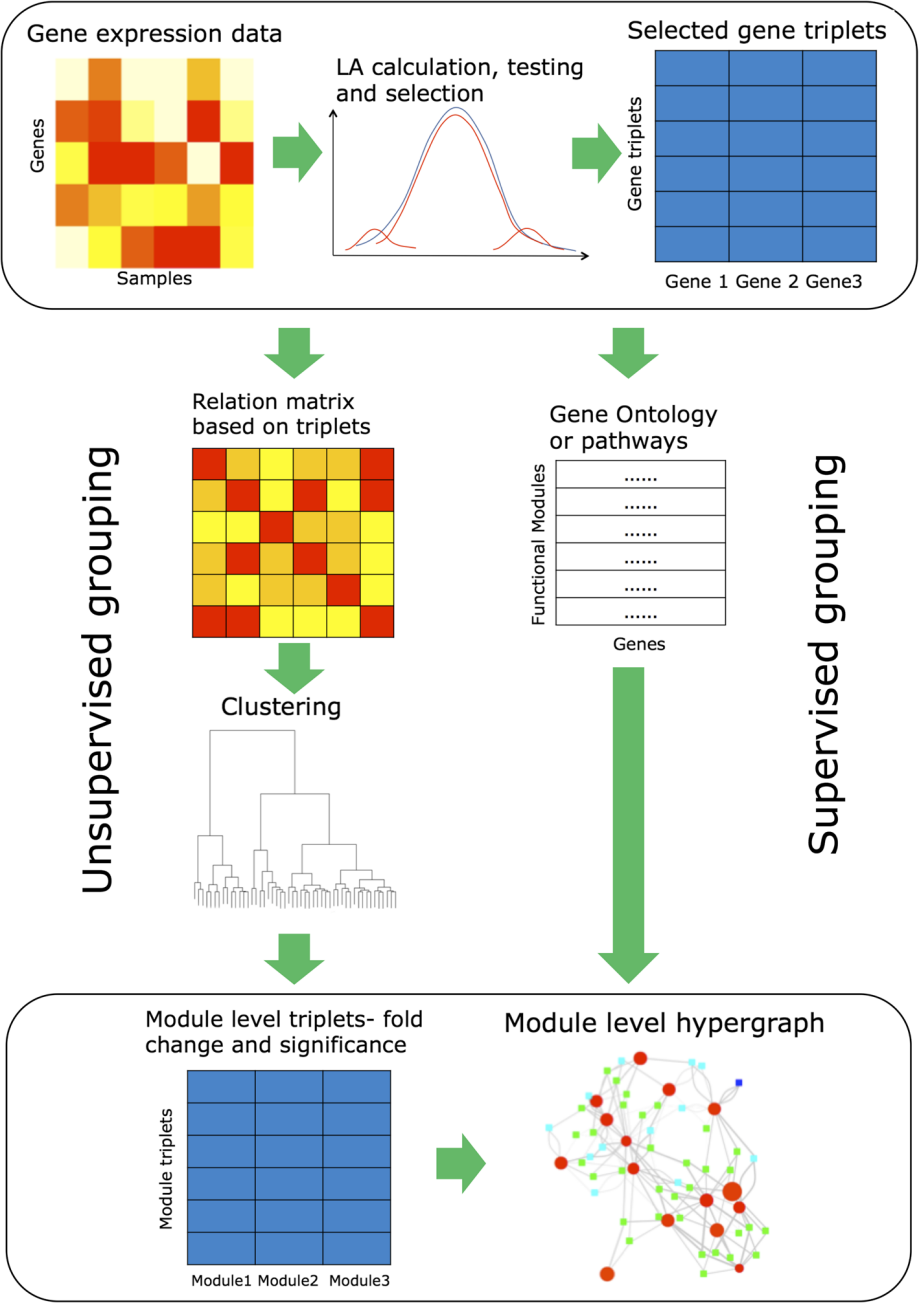
The flow chart of the analysis

We utilized Liquid Association (LA) [5], which is the most computationally tractable among the methods for the initial gene-level ternary relationship quantification. Screening procedures using mixture models are conducted to ensure the LA accurately detects significant ternary correlation, according to [11]. Two approaches of grouping genes are then introduced, one of which involves a new clustering procedure based on ternary relations. Using these approaches, three-way interaction hypergraphs are constructed. The workflow of our analysis is demonstrated in Fig. 1. We applied our methods to two real datasets, the TCGA human cutaneous melanoma dataset [17] and the the yeast cell cycle dataset [18]. For both datasets, module-level three-way interaction networks were obtained, exhibiting relations that conform to existing knowledge, as well as pointing to new and plausible dynamic correlations.

## Results

### Human cutaneous melanoma dataset

We applied our methods, which require an *n*×*m* matrix as input, to the Cutaneous Melanoma RNA-seq dataset from The Cancer Genome Atlas (TCGA) [17]. The original dataset contains 20,530 genes and 474 samples (*m*=474). After excluding genes with more than ten percent zero values, 15,274 genes (*n*=15,274) were retained for testing our method.

Each gene was first normalized using the normal score transformation as recommended in [5]. Before calculating LA using Eq. 1 in “Methods” section, to satisfy the sufficient condition described in the first sub-section of “Quantifying the ternary relationship”, we calculated the variance covariance matrix of all genes, obtaining a bell-shaped unimodal empirical distribution of pairwise correlations with mean *μ*≈0 and standard deviation *σ*. Then, only pairs with a correlation contained in the interval (*μ*−*cσ*,*μ*+*cσ*) were considered in LA calculation, where *c* is a small constant. In other words, no triplet would contain a pair having a correlation coefficient more than *μ*+*cσ* or less than *μ*−*cσ*. For this dataset, we have (*μ*−0.5*σ*,*μ*+0.5*σ*)=(−0.079,0.112).

Applying Eq. 1 along with the permutation selection using Eq. 2 in the “Methods” section, a total of 203,330,269 triplets were selected for this dataset at *fdr*=0.01. Given the information of the selected triplets, both supervised grouping and unsupervised grouping were conducted. We employed the GO term functional modules [19] as the external information for supervised grouping. A subset of informative GO terms with minimal overlap were selected using the procedure described in [20]. The count matrix *A* and its correlation matrix *C*, described in the third sub-section of “Selecting gene modules using supervised and unsupervised approaches”, were calculated, and the clusters were chosen using the technique proposed by [21], with the minimum cluster size of 100. The final numbers of modules for the two approaches were 423 and 77, respectively.

**Fig. 2:**
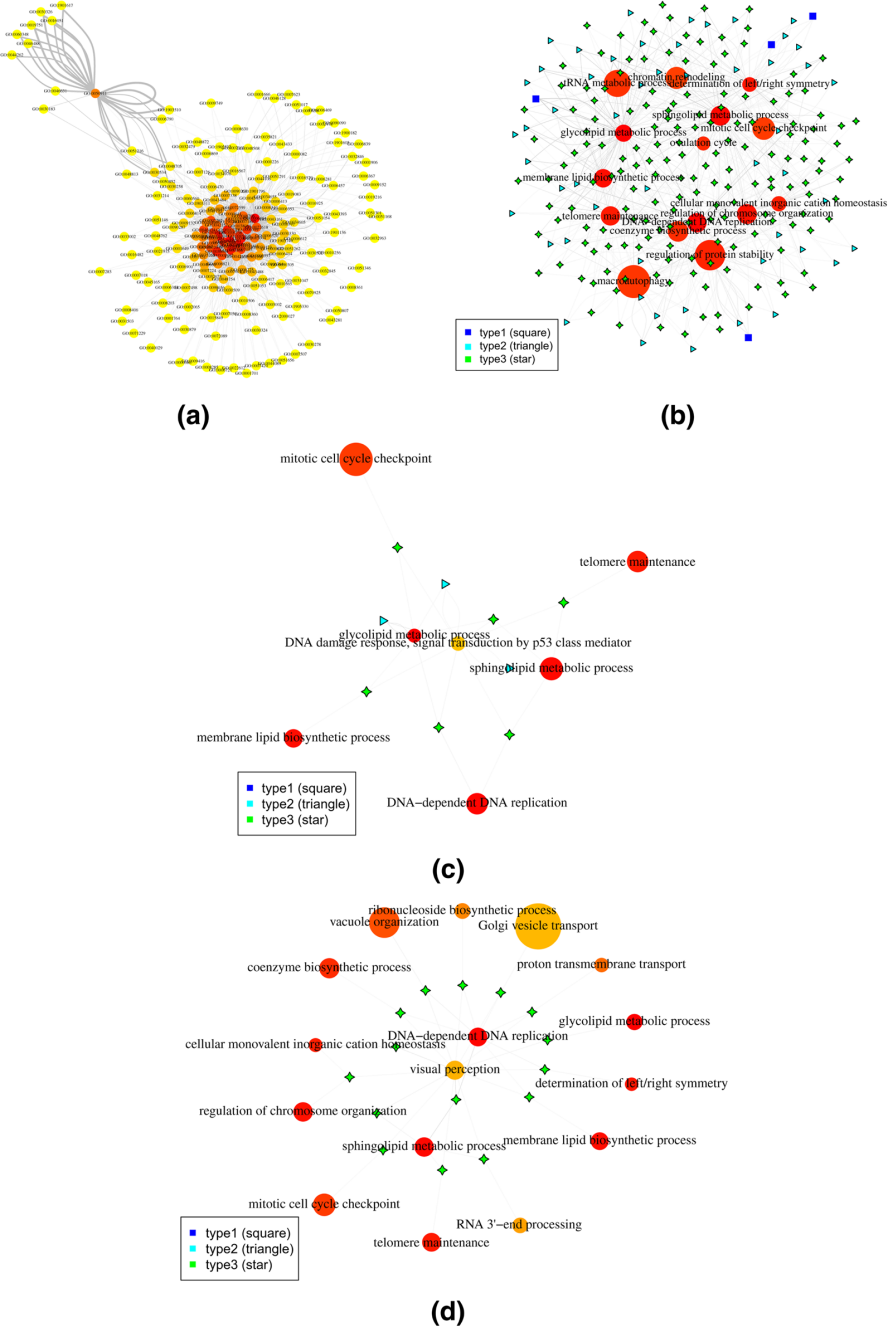
Visualization of the hypergraph for the TCGA melanoma dataset with supervised grouping. **a** The plot of the entire network, where hyperedges were reduced to binary edges for visualization; **b** Detailed plot of the top 15 most connected vertices; **c** Sub-hypergraph centered at the module “DNA damage response, signal transduction by p53 class mediator”; **d** Sub-hypergraph centered at the module “DNA dependent DNA replication”. Vertex colors reflect the degree of connections, with more connected more red and less connected more yellow. Vertex sizes reflect the module sizes. The width of each edge is the rescaled edge weight. Three types of hypergraph edges are presented: type 1 edge connects only one vertex; type 2 edge connects two different vertices; and type 3 edge connects three different vertices

Two three-uniform hypergraphs were constructed corresponding to the two grouping results. For the hypergraph with supervised grouping, edges were filtered with a minimum fold change of 2. The median number of connections for all the nodes involved in the graph is 4 (Fig. 2a). Figure 2b is a more detailed sub-hypergraph with the top 15 most connected vertices. The vertex color represent the number of connections of a vertex, with the most connected in red and least connected in yellow. The sizes of the vertices represent the number of genes in each module. Three types of edges were annotated corresponding to the three cases discussed in the last sub-section of “Methods” “Constructing the module-level hypergraph”. The width of edges are proportional to their weights.

Among the top 15 most connected nodes, 5 were related to the cell cycle and DNA metabolism, indicating the tight regulation in cellular reproduction in cancer cells. Three were related to lipid metabolism, the regulation of which has been shown to play critical roles in cancer progression and metastasis [22, 23], however traditional correlation-based methods haven’t shown their prominent role in expression dynamics.

To facilitate detailed examination, we examined sub-hypergraphs centered around a given vertex, together with all vertices directly connected with this vertex. As an example, Fig. 2c shows the sub-hypergraph centered at the functional module “DNA damage response, signal transduction by p53 class mediator”. Its connections involve both cell cycle modules and lipid metabolism modules. The role p53 pathway plays in lipid metabolism was only recently established [24]. Together with the fact that three lipid metabolism modules were among the most highly connected vertices, the results suggested a prominent role of lipid metabolism pathways, including sphingolipid, glycolipid, and membrane lipid metabolism, in human cutaneous melanoma development. Interestingly, there were three type 2 hyperedges in the subgraph, two of which each had two connections to the p53 module, meaning an excess of gene triplets having two genes falling into this module.

As another example, (Fig. 2d) shows the sub-hypergraph centered at the functional module “DNA dependent DNA replication”, which is a key process in cancer cell division. Besides other cell cycle related modules, those connected with “DNA dependent DNA replication” included several modules of organization of cellular organelles, as well as several modules of transport, indicating the tight control of the cell cycle process involves much of conditional correlations between genes. Interestingly, the function “visual perception” was at a central position in this subgraph, sharing 10 hyperedges with “DNA dependent DNA replication”. In the following analyses, we further explored the gene level relations of some of the hyperedges.

**Fig. 3:**
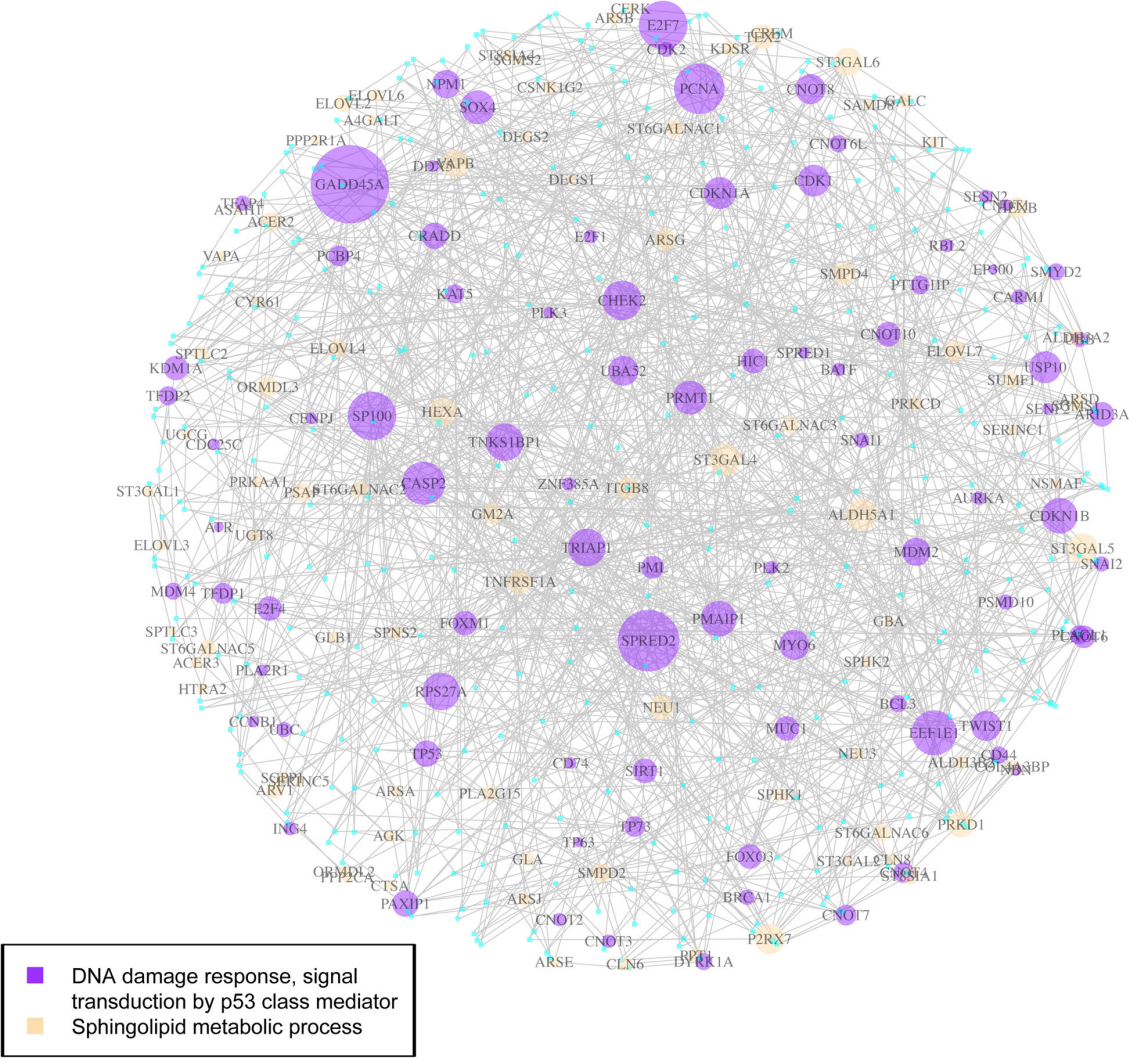
Visualization of the gene-level hypergraph of the triplet “DNA damage response, signal transduction by p53 class mediator”, “DNA damage response, signal transduction by p53 class mediator”, and “sphingolipid metabolic process”. Vertex sizes reflect the degree, with more connected nodes larger. All gene-level hyperedges are type 2 edges

Figure 3 shows the gene-level details of a triplet formed by the two modules “DNA damage response, signal transduction by p53 class mediator” and “sphingolipid metabolic process” in Fig. 2c. For each triplet, two of the three genes are from “DNA damage response, signal transduction by p53 class mediator” and the other one belongs to “sphingolipid metabolic process”, thus all gene-level hyperedges in Fig. 3 are type 2 edges. Among the genes belonging to the p53 pathway, several were prominent in terms of the number of hyperedge connections. For example, GADD45A (Growth Arrest And DNA Damage Inducible Alpha) is induced by stressful growth arrest or DNA-damaging agent treatment. The gene mediates stress response by activating the p38/JNK pathway. Down-regulation of the gene increases the chemosensitivity of melanoma [25]. SPRED2 (Sprouty Related EVH1 Domain Containing 2) is a member of the Sprouty/SPRED family of proteins that regulate growth factor-induced activation of the MAPK cascade, an apoptosis enhancer in melanoma [26]. E2F7 (E2F Transcription Factor 7) is among the transcription factors that regulate cell cycle progression, DNA damage repair and genomic stability. It plays a role in multiple types of cancers [27].

Among the highly connected genes that belong to the sphingolipid metabolism pathway, three were sialyltransferases - ST3GAL4 (ST3GAL4 ST3 beta-galactoside alpha-2,3-sialyltransferase 4), ST3GAL5, and ST3GAL6. Increased level of ST3GAL4 mRNA in renal cell carcinoma (RCC) has been shown to be associated with favorable prognosis [28]. In hepatocellular carcinoma (HCC), the microRNA miR-26a can reduce tumor growth by suppressing the Akt/mTOR pathway through targeting ST3GAL6 [29]. The role of the sialyltransferases in melanoma is yet to be elucidated.

Beside the sialyltransferases, other highly connected sphingolipid metabolism genes include ALDH5A1 (aldehyde dehydrogenase 5 family member A1), the reduced expression of which in high-grade serous ovarian cancer (HGSOC) causes the accumulation of hydroxybutyric acid (HBA) [30], and HEXA (hexosaminidase subunit alpha), the protein level of which was found to be increased among metastatic uveal melanoma [31].

**Fig. 4:**
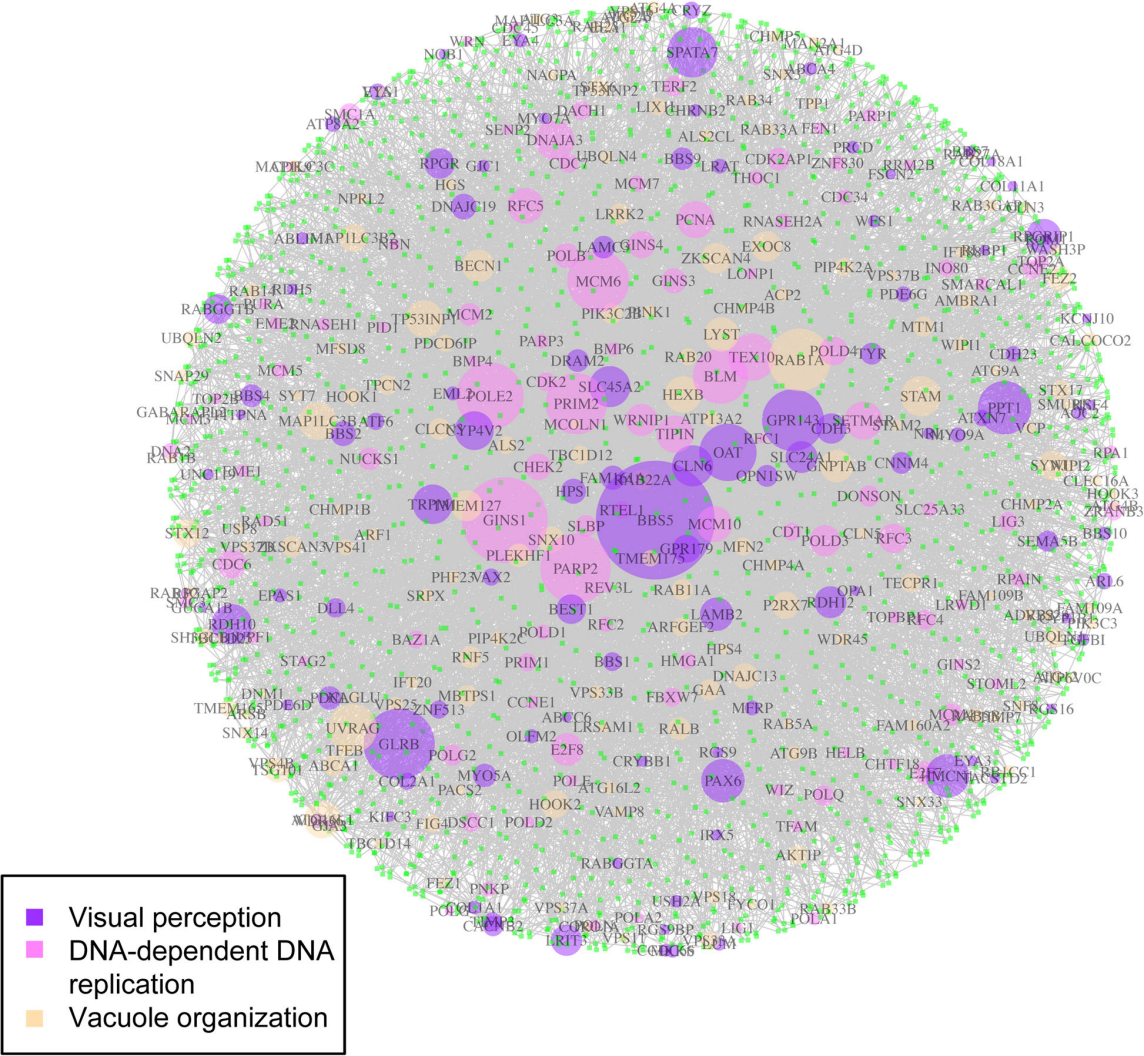
Visualization of the gene-level hypergraph of the triplet “visual perception”, “DNA-dependent DNA replication”, and “vacuole organization”. Vertex sizes reflect the degree, with more connected nodes larger. All gene-level hyperedges are type 3 edges

We further examined the gene-level hypergraph of the triplet “visual perception”, “DNA-dependent DNA replication”, and “vacuole organization” (Fig. 4). Here we focus on the discussion on genes from the first GO term “visual perception”, as the other two play obvious roles to melanoma development. The most highly connected gene, BBS5(Bardet-Biedl syndrome 5) has not been fully characterized, and its role in cancers not been well studied. Among other highly connected genes belonging to “visual perception”, GLRB (Glycine Receptor Beta) is among the ion channel genes that is associated with the clinical outcome in breast cancer [32]. GPR143 (G protein-coupled receptor 143, or OA1), codes a protein for pigmentation. SNPs in this gene have been found to be associated with the level of skin pigmentation and sun tolerance [33]. The gene is highly expressed in retinal pigment epithelium, as well as in melanoma [34]. It is involved in melanoma cell migration through the RAS/RAF/MEK/ERK signaling pathway [35]. PPT1 (palmitoyl-protein thioesterase 1), is involved in the lipid-modified protein catabolism in lysosomal degradation. Targeting PPT1 blocks mTOR signaling, which reduces tumor growth of melanoma in mouse models [36].

**Fig. 5:**
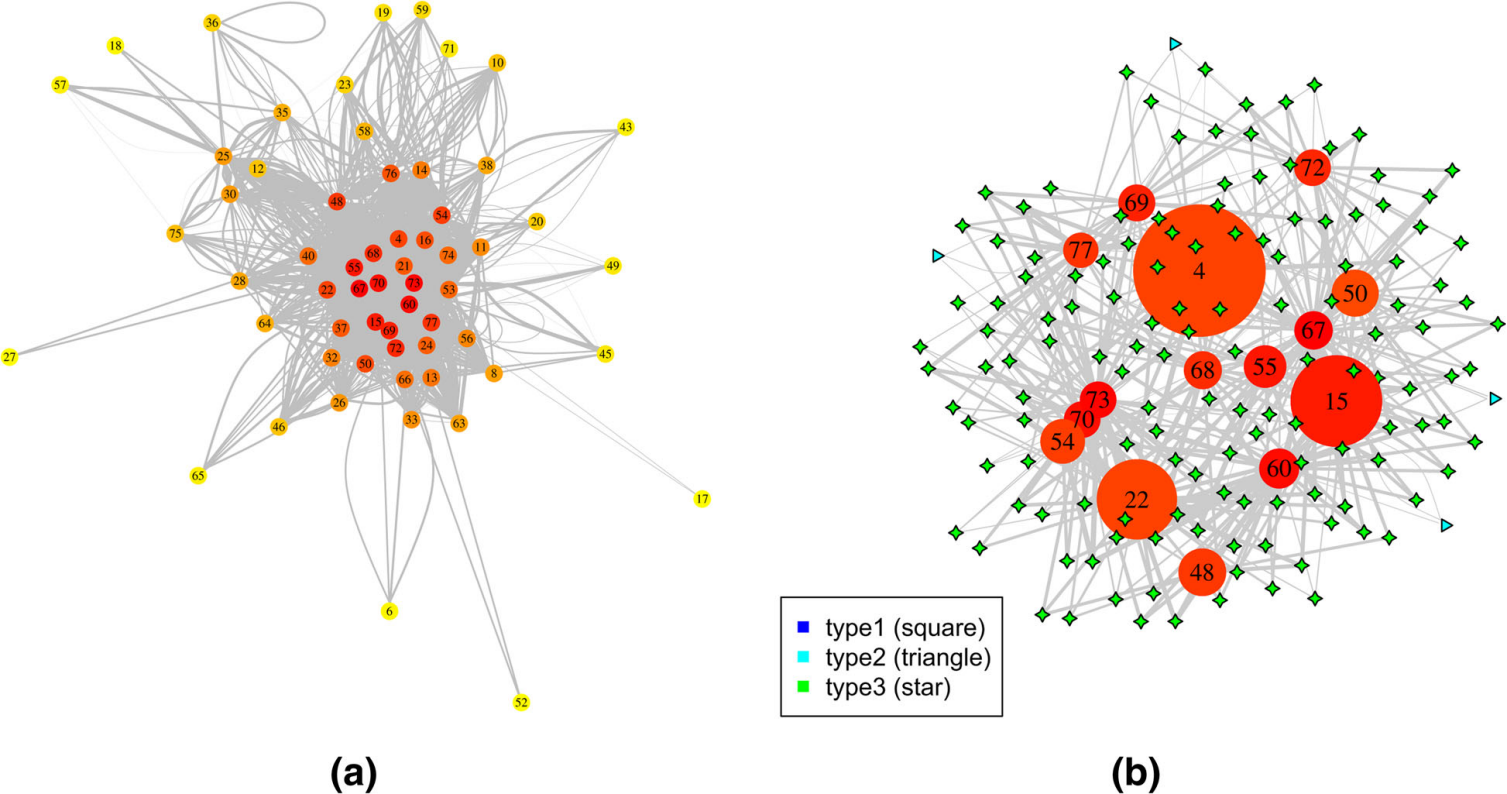
Visualization of the hypergraph for the TCGA melanoma dataset with unsupervised grouping. **a** The plot of the entire network. **b** Detailed plot of the top 15 most connected vertices. Vertex colors reflect the degree of connections, with more connected more red and less connected more yellow. Vertex sizes reflect the module sizes. The width of each edge is the rescaled edge weight. Three types of hypergraph edges are presented: type 1 edge connects only one vertex; type 2 edge connects two different vertices; and type 3 edge connects three different vertices

For the hypergraph with unsupervised grouping, edges were filtered with a minimum fold change of 10, which yielded a hypergraph with a median of 22 connections per node. Figure 5a is the plot of the entire hypergraph, and Fig. 5b is a more detailed sub-hypergraph with the top 15 most connected vertices. Figure settings are identical to those in the supervised case except for the vertex names. Similar to the supervised approach, the graph is also of scale-free structure, i.e. relatively few nodes were highly connected, while most nodes were connected to few other nodes.

With the unsupervised approach, functions of each cluster of genes were unknown. Thus, only the cluster IDs are shown in the plots. To further assess the meaning of each cluster, GO enrichment analysis was conducted to determine the relevant biological functions for the clusters using GOstats [37]. The corresponding gene set enrichment results for the top 15 most connected clusters are shown in Table 1. The gene set enrichment analysis was limited to GO biological processes with 5 to 500 genes. For each cluster, two significant gene set that included the most number of genes in the cluster, after manual removal of obvious overlapping biological processes, are shown in Table 1. The results largely agreed with the supervised grouping approach to some extent. Some of the terms were related to the cell cycle and lipid metabolism themes represented by the top 15 terms in the supervised approach, e.g. “double-strand break repair”, “actin filament bundle assembly”, “regulation of cytoskeleton organization”, “translational elongation”, and “steroid metabolic process”. At the same time, more terms in Table 1 point to some other general themes including stress response (e.g. “endoplasmic reticulum unfolded protein response” and “proteasome-mediated ubiquitin-dependent protein catabolic process”), small molecule metabolism (e.g. “cellular amino acid catabolic process” and “water-soluble vitamin metabolic process”), structure developments (e.g. “blood circulation” and “cell-cell adhesion via plasma-membrane adhesion molecules”), and signal transduction (e.g. “adenylate cyclase-activating G-protein coupled receptor signaling pathway” and “signal transduction by p53 class mediator”). The full list of the enrichment results are listed in Additional file 1.

**Table 1:** Enrichment analysis of the human dataset for the top 15 most connected clusters

Group label	Group size	Hyperedges involved	GOBPID	Term	*P*-value
67	109	210	GO:0030968	endoplasmic reticulum unfolded protein response	3.39E-05
			GO:0008015	blood circulation	3.81E-03
73	104	170	GO:0008202	steroid metabolic process	6.38E-04
			GO:0009063	cellular amino acid catabolic process	2.38E-03
60	114	142	GO:0007189	adenylate cyclase-activating G-protein coupled receptor signaling pathway	2.55E-05
			GO:0015698	inorganic anion transport	3.83E-04
70	104	120	GO:0072330	monocarboxylic acid biosynthetic process	3.21E-03
			GO:0019730	antimicrobial humoral response	6.04E-03
55	123	96	GO:0031349	positive regulation of defense response	3.26E-03
			GO:0043161	proteasome-mediated ubiquitin-dependent protein catabolic process	7.11E-03
15	266	83	GO:0006022	aminoglycan metabolic process	1.70E-03
			GO:0090066	regulation of anatomical structure size	2.31E-03
69	105	81	GO:0006302	double-strand break repair	1.47E-03
			GO:0001701	in utero embryonic development	2.93E-03
72	104	77	GO:0098742	cell-cell adhesion via plasma-membrane adhesion molecules	5.97E-03
			GO:0031365	N-terminal protein amino acid modification	7.54E-03
68	108	76	GO:0048608	reproductive structure development	2.43E-03
			GO:0008015	blood circulation	3.81E-03
77	100	71	GO:0051493	regulation of cytoskeleton organization	1.02E-03
			GO:0060560	developmental growth involved in morphogenesis	3.71E-03
48	138	68	GO:0051640	organelle localization	2.60E-04
			GO:0006767	water-soluble vitamin metabolic process	2.21E-03
54	129	56	GO:0051017	actin filament bundle assembly	1.26E-03
			GO:0009100	glycoprotein metabolic process	3.50E-03
4	385	51	GO:0016569	covalent chromatin modification	3.52E-03
			GO:0072331	signal transduction by p53 class mediator	4.46E-03
22	233	51	GO:1901655	cellular response to ketone	2.65E-03
			GO:0010035	response to inorganic substance	8.21E-03
50	136	49	GO:0006414	translational elongation	1.45E-03
			GO:0002791	regulation of peptide secretion	4.27E-03

For each cluster, the enriched term that include the most number of genes in the cluster is shown

In the unsupervised approach, genes are grouped based on their LA relation patterns with other genes. Thus genes annotated to different biological processes can be grouped together. At the same time, genes in the same biological pathway could have diverse expression activities, and be separated into different groups. Thus unsupervised approach can complement the supervised approach, painting a more complete picture of the global dynamic correlation activities.

### Yeast cell cycle dataset

We also applied our methods to the yeast cell cycle microarray dataset [18]. The yeast dataset contains 6178 genes (*n*=6178), and 73 samples in four short time series and 4 control samples (*m*=77). For the yeast cell cycle dataset, we have restricted the pairwise correlation interval (*μ*−*σ*,*μ*+*σ*)=(−0.180,0.210), and a total of 3,782,460 triplets were selected for this dataset at *fdr*=0.2. Again both supervised grouping and unsupervised grouping were conducted. Given the smaller number of genes, for the dynamic tree cut method we used a minimum cluster size of 20. The final numbers of modules for the two approaches were 251 and 53, respectively.

**Fig. 6:**
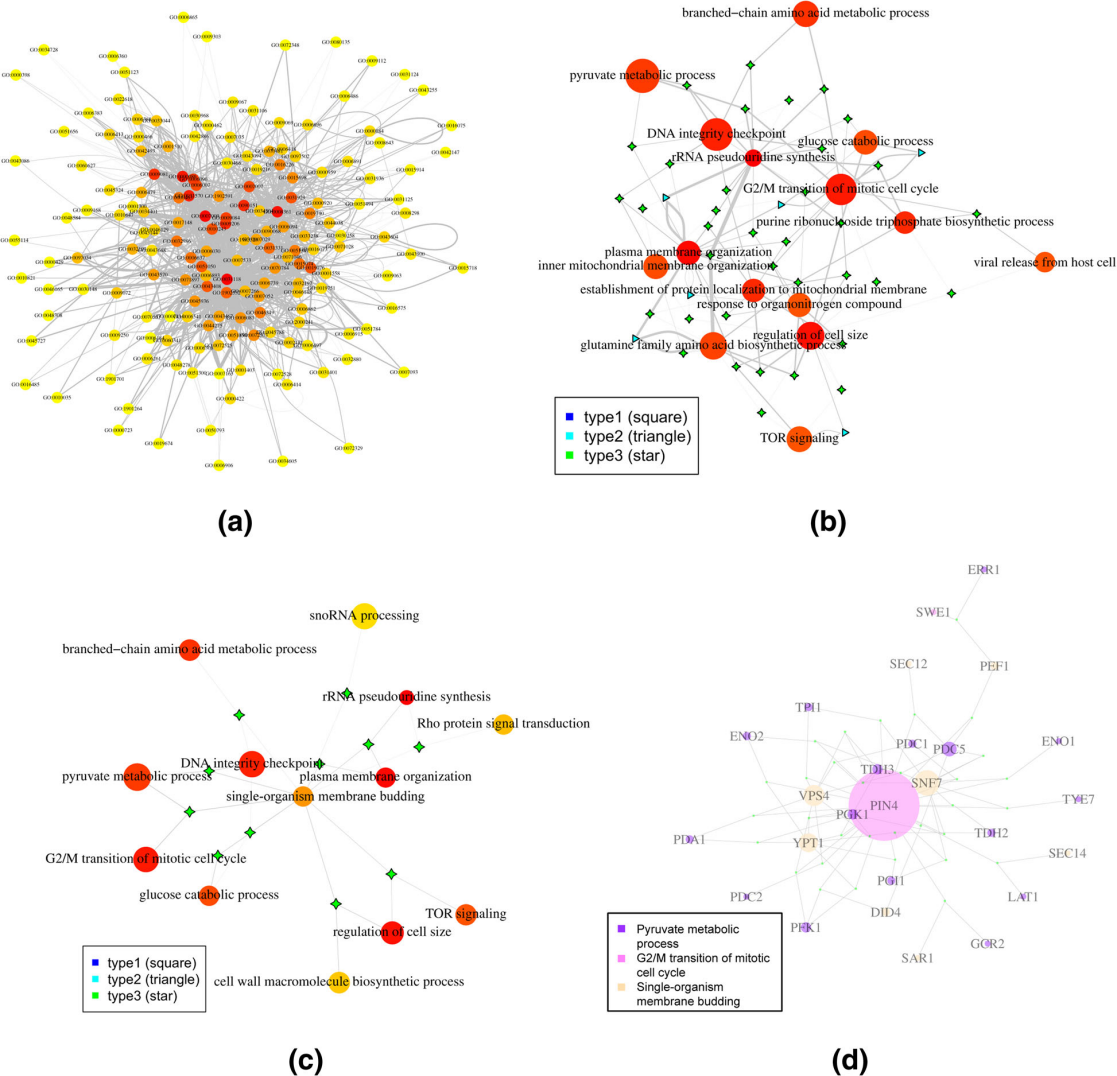
Hypergraph of the yeast cell cycle dataset with supervised grouping. **a** The plot of the entire network; **b** Detailed plot of the top 15 most connected vertices; **c** Sub-hypergraph centered at the module Single organism membrane budding; **d** Gene level hypergraph for the module triplet “single-organism membrane budding”, “G2M transition of mitotic cell cycle”, and “pyruvate metabolism”. For the module-level hypergraph, vertex sizes reflect the degree of connections, with more connected more red and less connected more yellow. Vertex sizes reflect the module sizes. The width of each edge is the rescaled edge weight. Three types of hypergraph edges are presented: type 1 edge connects only one vertex; type 2 edge connects two different vertices; and type 3 edge connects three different vertices. For the gene-level hypergraph, vertex sizes reflect the degree, with more connected nodes larger

For the hypergraph with supervised grouping, edges were filtered with a minimum fold change of 8. The median number of connections for all the nodes involved in the graph is 4 (Fig. 6a). Figure 6b is a more detailed sub-hypergraph with the top 15 most connected vertices. Beside some cell-cycle related modules, the majority of the top 15 connected modules were related to small molecule metabolism and membrane organization (Fig. 6b). Although the dataset was generated from synchronized cell cycles, the results suggested that much of the conditional correlations happened in metabolism, which was consistent with findings of the original LA paper [5].

Figure 6c shows an example sub-hypergraph centered at the functional module “Single organism membrane budding”. Besides membrane and cell wall organization terms, most of the terms were related to small molecule metabolism terms. Figure 6d shows the gene-level details of the dynamic correlations of the triplet “Single organism membrane budding”, “G2M transition of mitotic cell cycle”, and “pyruvate metabolism”. It is interesting that PIN4 (YBL051C) played a central role in the graph. PIN4 functions in G2/M phase transition and DNA damage response. Its expression level didn’t simply track the progression of cell cycle. In fact it was not one of the periodic genes found in the original study of [5]. Hence its central role in the gene-level graph was not caused by it being a proxy indicator of the cell cycle. Rather, PIN4 expression tend to be lower at the start of three of the four time series, except in the cdc15 time series. The cell cycle synchronization was conducted by blocking the cells at a certain phase of the cell cycle, which understandably put the cells in a stress state and cause irregularities in metabolism. The expression of PIN4 likely represents part of the recovery mechanism to normal growth state.

**Fig. 7:**
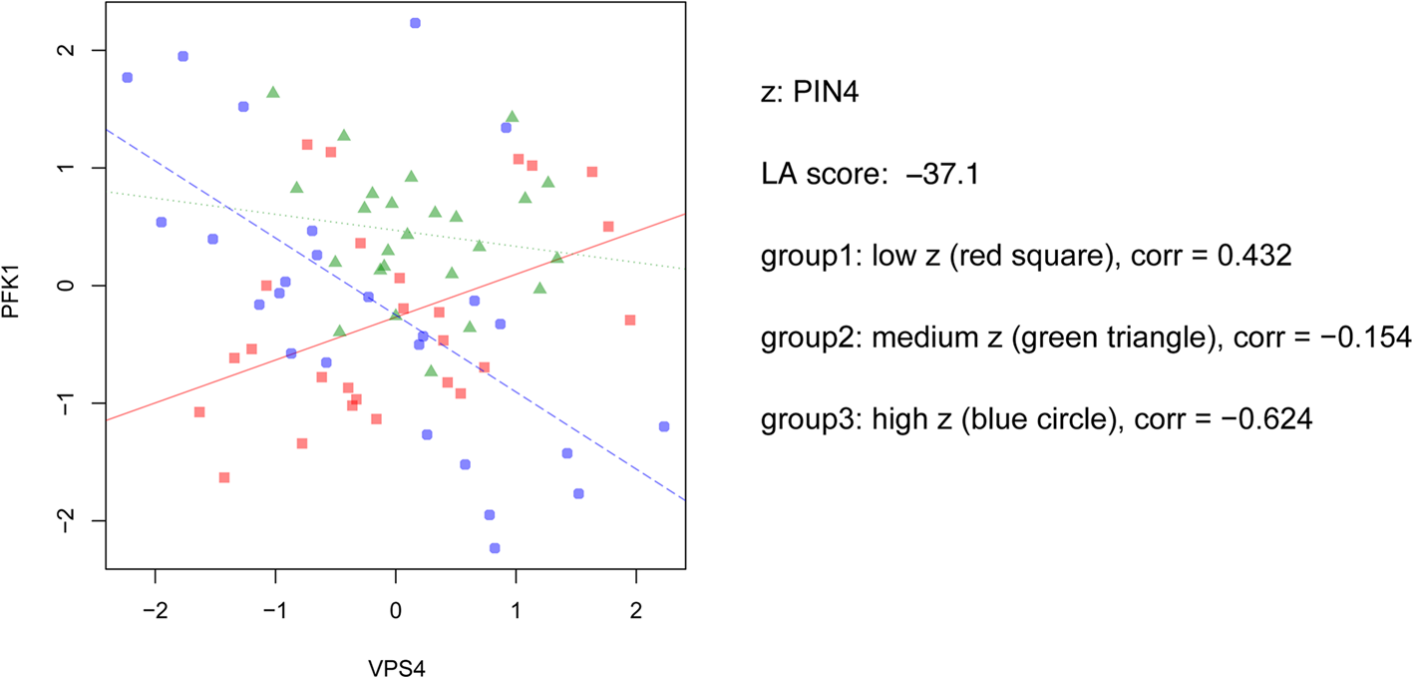
An example triplet of yeast genes. The 2-D scatter plot of expression values from PFK1 and VRS4 is given, where PIN4 serves as the “scouting gene” z. The points are divided into three groups according to the expression level of z, with low level the first 1/3 percentile, medium level the middle 1/3 percentile, and high level the last 1/3 percentile. Three lines (red solid, green dotted, and blue dashed) denote the corresponding linear regression lines of PFK1 over VRS4, given the three levels of PIN4

Conditioned on the level of PIN4, the correlation pattern changed between genes involved in budding and pyruvate metabolism. Three of the budding genes were prominent, SNF7 (YLR025W, vacuolar-sorting protein), VPS4 (YPR173C, vacuolar protein sorting-associated protein) and COX12 (YFL038C, cytochrome c oxidase subunit). Both SNF7 and VPS4 are involved in protein sorting [38], and both VPS4 and COX12 are involved in energy production [39]. Pyruvate is at a key intersection in metabolic network. It can be converted into carbohydrates, fatty acids, amino acid, or ethanol [40]. A number of the genes involved in pyruvate metabolism show dynamic correlations, either between themselves, or with the budding genes, indicating a change of production and utilization of pyruvate that is dependent on the cells’ recovery from the unnatural blockage state as indicated by PIN4 levels. An example gene pair PFK1 (YGR240C, Alpha subunit of heterooctameric phosphofructokinase) and VPS4 are shown in Fig. 7. We can observe a strong inverse correlation between the low-PIN4 and high-PIN4 states.

**Fig. 8:**
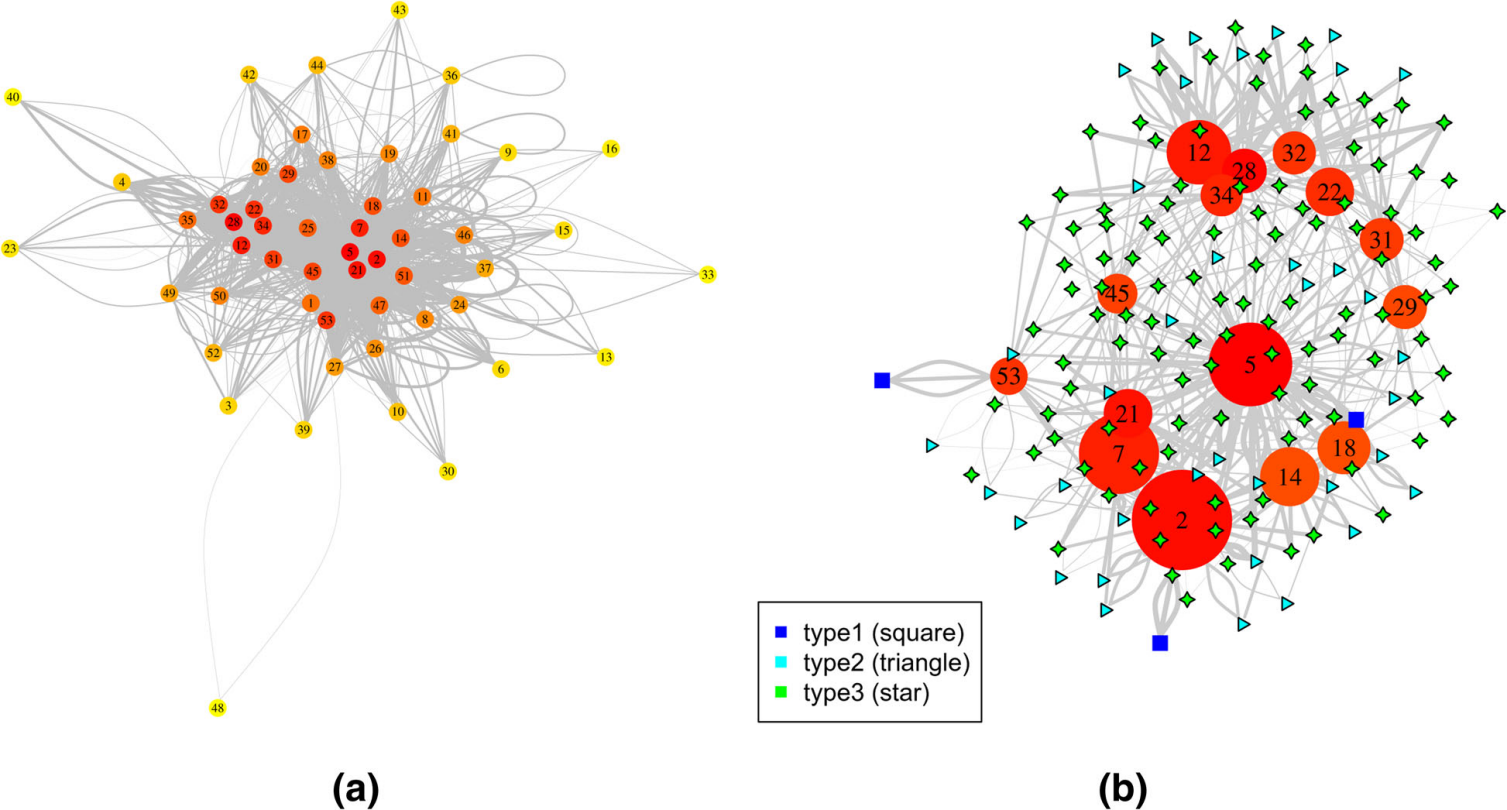
Visualization of the hypergraph for the yeast dataset with unsupervised grouping. **a** The plot of the entire network; **b** Detailed plot of the top 15 most connected vertices. Vertex sizes reflect the degree of connections, with more connected more red and less connected more yellow. Vertex sizes reflect the module sizes. The width of each edge is the rescaled edge weight. Three types of hypergraph edges are presented: type 1 edge connects only one vertex; type 2 edge connects two different vertices; and type 3 edge connects three different vertices

For the hypergraph with unsupervised grouping, edges were filtered with a minimum fold change of 4, which yielded a median of 20 connections per node involved in the graph (Fig. 8a). Figure 8b is a more detailed sub-hypergraph with the top 15 most connected vertices. The enrichment results for the top 15 most connected clusters are shown in Table 2. Four of the top 15 clusters were dominated by cell cycle processes (e.g. “mitotic cell cycle” and “regulation of cytokinesis”). In addition, three of the terms were dominated by protein synthesis (e.g. “translation” and “ribosome biogenesis”). The other clusters were mostly dominated by small molecule metabolism/transport (e.g. “oxidation-reduction process” and “organic acid catabolism”), especially in relation to carbohydrate and energy (e.g. “regulation of glycogen biosynthetic process” and “monosaccharide metabolic process”). These results largely agreed with those from the supervised approach. The full list of the enrichment test can be seen in Additional file 2.

**Table 2:** Enrichment analysis of the yeast dataset for the top 15 most connected clusters

Group label	Group size	Hyperedges involved	GOBPID	Term	*P*-value
5	180	432	GO:0005979	regulation of glycogen biosynthetic process	1.34E-03
			GO:0016052	carbohydrate catabolic process	5.17E-03
28	46	85	GO:0032543	mitochondrial translation	2.28E-03
			GO:0072329	monocarboxylic acid catabolic process	6.64E-03
2	237	82	GO:0030154	cell differentiation	2.15E-03
			GO:0030435	sporulation resulting in formation of a cellular spore	5.63E-03
21	60	69	GO:0006259	DNA metabolic process	1.10E-09
			GO:1903047	mitotic cell cycle process	7.00E-06
12	114	61	GO:0022613	ribonucleoprotein complex biogenesis	4.25E-13
			GO:0034660	ncRNA metabolic process	1.97E-10
7	168	59	GO:0005996	monosaccharide metabolic process	7.62E-03
34	38	55	GO:0016236	macroautophagy	4.56E-03
			GO:0048193	Golgi vesicle transport	9.15E-03
22	59	52	GO:0055114	oxidation-reduction process	3.84E-05
			GO:0071822	protein complex subunit organization	2.70E-04
32	41	48	GO:0042254	ribosome biogenesis	2.77E-15
			GO:0034470	ncRNA processing	1.02E-09
53	20	48	GO:0032465	regulation of cytokinesis	1.28E-03
			GO:0045839	negative regulation of mitotic nuclear division	9.30E-03
31	43	40	GO:0000278	mitotic cell cycle	5.15E-07
			GO:0007010	cytoskeleton organization	3.73E-04
45	28	39	GO:0016054	organic acid catabolic process	1.21E-03
			GO:0015074	DNA integration	2.56E-03
29	44	36	GO:0034637	cellular carbohydrate biosynthetic process	2.43E-03
			GO:0055114	oxidation-reduction process	6.09E-03
14	96	35	GO:0006412	translation	1.64E-32
			GO:0022613	ribonucleoprotein complex biogenesis	2.99E-16
18	75	35	GO:0007030	Golgi organization	5.04E-04
			GO:0051784	negative regulation of nuclear division	2.15E-03

For each cluster, the enriched term that include the most number of genes in the cluster is shown

## Discussion

The method involves several hyper-parameters. To calculate the LA score of a triplet, we tried to create a sufficient condition according to Theorem 1 of [11], to discover “real” dynamic correlation. It requires that any pair of genes should not be linearly associated in a triplet. Thus, the threshold *c* is a hyper-parameter controlling how strict the user wants to obey the sufficient condition. If *c* is too small, one can hardly find triplets as few pairs would have strictly zero correlation from the sample correlation perspective. However, if c is too large, the sufficient condition for real LA would be violated too much, leading to false discovery for the entire downstream analysis. Therefore, the constant *c* can be set partially heuristically to decide the trade-off. On the other hand, the sample size of the data determines the sampling variation of the Pearson’s correlation between pairs of genes that are truly uncorrelated. The TCGA melanoma data contains 474 samples. Based on Fisher’s transformation of the Pearson’s correlation, if two genes are truly uncorrelated, by random sampling variation, the standard deviation of their correlation value is 0.046. Thus if two genes are uncorrelated, the 95% confidence interval (CI) of their sample correlation is (-0.09, 0.09) without adjusting for multiple testing. For this dataset, as the actual average of correlation values was not exactly zero, we used *c*=0.5 and the corresponding interval of (-0.079, 0.112), which roughly matched that of the 95% CI. Similarly, the yeast cell cycle data contains 77 samples, which means if two genes are truly uncorrelated, by random sampling variation, the standard deviation of their correlation value is 0.114. Hence the 95% CI of the sample correlation coefficient if two genes are uncorrelated is (-0.22, 0.22). We used *c*=1 that yielded an interval of (-0.18, 0.21), which again roughly matched the 95% CI while allowing the mean to be non-zero.

Another important parameter is the selection of fold change threshold to generate the module-level graph. As the fold change threshold increases, more connection information would be lost, though the hypergraph would be less dense and easier to investigate. Hence, similar to the correlation threshold *c*, the fold change threshold is also a user-specified parameter to balance the trade-off between information cleanness and completeness. In practice, we selected fold change thresholds such that the median of the degrees of the modules was 4 in the supervised case, where hundreds of modules were involved. For the unsupervised results, as roughly 50 modules were involved, we selected fold change thresholds such that the median degree was around 20. These choices made it easy to visually inspect the resulting graphs.

In this manuscript, we proposed two routes of data analysis, the supervised approach and the unsupervised approach. The supervised approach relies on existing annotations of the genes to determine the modules, while the unsupervised approach uses the gene-level connection patterns to group genes into modules. As we have seen in the results, the two approaches generated results that largely agree, while each provided insights that complement the other approach. The supervised approach was generally easier to interpret. It allowed us to focus on important biological processes, such as the p53 pathway in the melanoma data. For a poorly annotated species, the unsupervised approach will help group genes that share similar LA relations. If genes are poorly annotated, this grouping can potentially shed light on their functional relations, and may help their functional annotation based on other genes in the same module that are well annotated.

## Conclusions

We presented a method to examine dynamic correlations in an unbiased manner at the transcriptomic scale. It uses an inference framework to defend against false positives, and reduces the large amounts of triplets into a manageable hypergraph that can be visually examined relatively easily. Complimenting existing correlation-based and partial correlation-based network construction methods, the new method provides a useful tool for users to study dynamic relations in gene expression profiling datasets.

## Methods

### Quantifying the ternary relationship

The input of our analysis is an ordinary *n*×*m* gene expression data matrix, with rows representing genes and columns representing specific samples. The ternary relationship is quantified by the statistic Liquid Association (LA) proposed in [5]. The LA statistic measures the extent to which the correlation of a pair of variables (*X,Y*) depends on the value of a third variable *Z*. Thus, the pairwise correlation is dynamic in the sense that it is affected dynamically according to the third variable. Based on this property, the LA statistic is therefore a suitable tool to quantify the ternary relationship for triplets of variables.

Specifically, according to [5], suppose we are interested in measuring the ternary correlation among *X*, *Y* and *Z*. Without loss of generality, we can regard the ternary correlation as the dynamic pairwise correlation between *X* and *Y* given the third variable *Z*. The LA statistic is three-way symmetric regardless which variable is treated as the conditioning variable, or “scouting gene”. Now let *g*(*Z*) be the conditional expectation of the correlation between *X* and *Y*, namely, *g*(*Z*)=*E*_*X,Y*_(*XY*|*Z*). Then, the LA statistic is defined as the expected changes of the correlation between *X* and *Y*: *LA*(*X,Y*|*Z*)=*E*_*Z*_(*g*^′^(*Z*)). When the variables are normalized with mean zeros, it is proved in [5] that *LA*(*X,Y*|*Z*)=*E*(*XYZ*), which means the LA statistic of *X* and *Y* given *Z* is just the expectation of the product *XYZ*. Therefore, the LA statistic can be estimated simply by calculating the sample mean of the product *XYZ*:

1 $$ E(XYZ)=\frac{1}{m}\Sigma_{i=1}^{m}X_{i}Y_{i}Z_{i},  $$

where *m* is the dimension of the variables. Note that following this definition, LA is invariant of which variable (*X*, *Y*, or *Z*) we treat as the dynamic correlation condition, hence gives a measure of the ternary correlation.

Straightforward as the LA is, [11] points out that for the quantity *E*(*XYZ*) to reflect the true dynamic correlation of *X* and *Y* given *Z*, certain conditions must be met. They therefore developed the Modified Liquid Association (MLA) statistic to detect the dynamic correlation more accurately, which incurs a much higher computing cost. Also, in [11], the authors proved that the MLA of *X* and *Y* given *Z* (denoted as *MLA*(*X,Y*|*Z*)) is equivalent to *E*(*XYZ*) as well when certain conditions are satisfied (Theorem 1, [11]). These conditions include the normality of the “third variable” *Z* and the distributions of *X*|*Z* and *Y*|*Z* have constant mean and variance.

In our analysis, the *n*×*m* gene expression data matrix is normalized using normal score transformation for every row following [5], and we are interested in the ternary correlation among three variables. In the initial phase of selecting related gene triplets, which specific variable serves as the dynamic condition is less important. Also, Li’s original approach is computationally better suited for transcriptome-scale scans. Thus, the invariant property regarding the dynamic condition variable of *E*(*XYZ*) is desirable. To preserve this property, we restrict the mutual pairwise correlations within a triplet to be small, creating a sufficient condition for Theorem 1 in [11]. To see how this is achieved, if *X*, *Y* and *Z* are marginally normally distributed, and all the three pairwise correlations, *corr*(*X,Y*),*corr*(*X,Z*) and *corr*(*Z,Y*)≈0, then *X*, *Y* and *Z* are three independent normal variables. Hence, if the sequence *U*_1_,*U*_2_,*U*_3_ is any permutation of *X,Y*,*Z*, then *E*(*U*_1_|*U*_3_)=*E*(*U*_1_)=0,*Var*(*U*_1_|*U*_3_)=*Var*(*U*_1_)=1,*E*(*U*_2_|*U*_3_)=*E*(*U*_2_)=0,*Var*(*U*_2_|*U*_3_)=*Var*(*U*_2_)=1, and $U_{3}\sim \mathcal {N}(0,1)$ are satisfied. Hence, the ternary correlation of a triplet (*X,Y*,*Z*) satisfies the condition in [11], and can be quantified by Eq. 1. Notice this requirement of low pairwise correlations also satisfy Li’s original setup of Liquid Association [5].

### Selecting significant triplets using permutation and mixture models

The ternary correlation is calculated gene by gene, namely for each gene *Z*, the sample product mean of *Z* and all possible gene pairs (*X,Y*) are calculated, for all the triplets satisfying the condition discussed in the above sub-section. We expect only a small portion of the triplets to have true ternary relationship. The ternary correlation of triplets with insignificant relationship approximately follow a normal distribution [11]. We employ a permutation procedure to estimate the parameters of the distribution.

To simplify our illustration, we define $\lambda _{(i,j)}^{(Z)}, (i,j)\in \{all\, possible\, pairs\,for\,Z\}$ as the ternary correlation associated with the given gene *Z* with the other two genes *X* and *Y* varying, and $\hat {\lambda }$ the sample product mean. The permutation selection is conducted as following: after calculating the ternary correlation $\hat {\lambda }^{(Z)}$ of all possible triplets for a gene *Z*, an empirical distribution of *λ*^(*Z*)^ is obtained. We then randomly permute the sample labels of *Z* and calculate all ternary correlations with all the {*X,Y*} pairs again, obtaining another empirical distribution of $\lambda ^{(Z^{*})}$, which is considered as the null distribution where X, Y and Z have no ternary relationship. We estimate the two densities of the distributions using the kernel density estimation technique [41]. Then, the ratio between the estimated permutation empirical density and the estimated actual empirical density, at a given value of ternary correlation *λ*, serves as the false discovery rate, i.e. the posterior probability that a *λ* at this value belongs to the null distribution:

2 $$ fdr^{(Z)}(\lambda)=\hat{f_{0}}^{(Z^{*})}(\lambda)/\hat{f}^{(Z)}(\lambda).  $$

Setting a small number of false discovery rate, say 0.1, we are able to obtain the corresponding threshold on the value of *λ*. Triplets with a false discovery rate lower than the threshold are selected. The calculation and selection procedure is repeated for every gene in the dataset. Finally we obtain the entire list of triplets with significant ternary correlation. We note that the *fdr* estimate doesn’t inflate in theory due to the large number of *Z*’s being considered, because the null density doesn’t change shape with more null *λ* values being calculated.

### Selecting gene modules using supervised and unsupervised approaches

As mentioned in the “Background” section, given the large amount of gene-level triplets, it is impractical to present the three-way interactions of the system as a whole. Therefore, it is necessary to “build up” the system structure to a gene module level by dividing genes in a dataset into different modules. To achieve this, two options are available. The first choice, which we refer to as supervised grouping, is to borrow external biological information such as gene functional modules from gene ontology (GO) terms [42]. We follow a procedure of selecting a subset of informative GO terms [43]. While some genes in the dataset may not be included in the functional modules, other genes may appear in more than one modules. In the first case, the genes are ignored since they do not contribute to module-level information according to the external information. In contrast, in the latter case, the duplication of a certain set of genes across multiple modules is preserved as the set of genes contribute to multiple module level information.

The alternative way of grouping genes is clustering based on the gene level hypergraph structure, which is correspondingly an unsupervised grouping approach. In this study we base our clustering on the marginal relations between pairs of genes. To utilize the information of ternary relationship provided by the triplets selected in the above sub-section “Selecting significant triplets using permutation and mixture models”, we first construct an *n*×*n* matrix *A* recording the number of involvement of pairs in triplets, where *n* is the total number of genes in the dataset. Specifically, for example, if a triplet of genes (*i,j*,*k*) is selected after the procedure described in the above two sub-sections “Quantifying the ternary relationship” and “Selecting significant triplets using permutation and mixture models”, then according to the existence of this ternary correlation, the elements *A*_*i,j*_,*A*_*j,i*_,*A*_*i,k*_,*A*_*k,i*_,*A*_*j,k*_,*A*_*k,j*_ are all added by one to receive a “count”. This counting procedure is repeated for the entire triplet list. Finally, the *A* matrix contains the amount of connections between any pair of genes when they jointly appear in a triplet. The diagonal elements of *A* are all set to zero since it is meaningless to consider self-connection here, and it is easy to see *A* is symmetric.

Given the matrix *A*, one can calculate the correlation matrix *C* for *A*, as it measures the similarity of the involvement in triplets among genes. Thus, using either the similarity matrix *C* or the corresponding distance matrix 1_*n*×*n*_−*C*, where 1_*n*×*n*_ is an *n*×*n* matrix with all elements equal to one, traditional distance-based clustering methods such as hierarchical clustering can be applied to cluster genes in the dataset. Essentially, the unsupervised grouping approach clusters genes according to their similarities of involvement in triplets.

### Constructing the module-level hypergraph

Using either supervised or unsupervised approach, the module memberships of genes are obtained. The next step is to replace each gene in the triplet list by its module label. In the case that some genes may have multiple module labels due to multi functionality in supervised grouping, the involved triplets are duplicated in order to preserve the multi-functional information as discussed in the above sub-section “Selecting gene modules using supervised and unsupervised approaches”.

At this stage, the module-level triplet list forms an edge list for a 3-uniform hypergraph, in which modules are vertices and triplets are hyperedges. The three-uniform hypergraph is undirected but weighted, as there can be many gene triplets establishing the connections between three modules. Consequently, three types of edges - those connecting three different modules, two different modules or only one module, exist in the hypergraph. These correspond to cases that the original three genes in a triplet are divided into three modules, two modules or a single module. Therefore, the 3-uniform hypergraph allows self-loops. Summing up all identical module-level triplets, the counts for each unique module-level triplet can serve as the weight of the corresponding hyperedge. Given the size difference of the modules, we transform the edge weights from simple counts to fold changes over the expected number of links if all edges are placed randomly. We then threshold the fold change to get a sparsely connected network.

## Additional files

Additional file 1 Enrichment test results for the TCGA melanoma data. List of full GO terms for all the 77 groups resulted from the unsupervised approach. (CSV 139 kb)

Additional file 2 Enrichment test results for yeast cell cycle data. List of full GO terms for all the 53 groups resulted from the unsupervised approach. (CSV 72 kb)
